# Inhibitory Effects of Gold and Silver Nanoparticles on the Differentiation into Osteoclasts In Vitro

**DOI:** 10.3390/pharmaceutics13040462

**Published:** 2021-03-29

**Authors:** Daye Lee, Wan-Kyu Ko, Seong Jun Kim, In-Bo Han, Je Beom Hong, Seung Hun Sheen, Seil Sohn

**Affiliations:** 1 CHA Bundang Medical Center, Department of Neurosurgery, CHA University, 59, Yatap-ro, Bundang-gu, Seongnam-si 13496, Gyeonggi-do, Korea; day03@chauniv.ac.kr (D.L.); wankyu@chauniv.ac.kr (W.-K.K.); ksj987456@chauniv.ac.kr (S.J.K.); hanib@cha.ac.kr (I.-B.H.); nssheen@cha.ac.kr (S.H.S.); 2 Department of Biomedical Science, CHA University, Bundang-gu, Seongnam-si 13496, Gyeonggi-do, Korea; 3 Department of Neurosurgery, Kangbuk Samsung Hospital, Sungkyunkwan University School of Medicine, Seoul 03181, Korea; jebeomhong@gmail.com

**Keywords:** gold nanoparticles, silver nanoparticles, osteoclast, bone marrow-derived macrophages, RANKL

## Abstract

Gold nanoparticles (GNPs) have been widely studied to inhibit differentiation into osteoclasts. However, reports of the inhibitory effects of silver nanoparticles (SNPs) during the process of differentiation into osteoclasts are rare. We compared the inhibitory effect of GNPs and SNPs during the process of differentiation into osteoclasts. Bone marrow-derived cells were differentiated into osteoclasts by the receptor activator of the nuclear factor-kappa-Β ligand (RANKL). The inhibitory effect of GNPs or SNPs during the process of differentiation into osteoclasts was investigated using tartrate-resistant acid phosphatase (TRAP) and actin ring staining. The formation of TRAP positive ^(+)^ multinuclear cells (MNCs) with the actin ring structure was most inhibited in the SNP group. In addition, the expression of specific genes related to the differentiation into osteoclasts, such as c-Fos, the nuclear factor of activated T-cells, cytoplasmic 1 (NFATc1), TRAP, and Cathepsin K (CTSK) were also inhibited in the SNP groups. As a result, the levels related to differentiation into osteoclasts were consistently lower in the SNP groups than in the GNP groups. Our study suggests that SNPs can be a useful material for inhibiting differentiation into osteoclasts and they can be applied to treatments for osteoporosis patients.

## 1. Introduction

Osteoporosis is caused by an imbalance between bone resorption by osteoclasts and bone formation by osteoblasts [1]. Specifically, bone resorption is necessary to maintain homeostasis for bone remodeling [1]. However, excessive resorption leads to osteoporosis [2,3]. Osteoclasts require several weeks to undertake the resorption of bone, whereas osteoblasts need several months to produce new bone. The relatively short process by osteoclasts can induce osteoporosis [4]. The interaction between osteoclasts and osteoblasts is important in the process of the bone remodeling. Osteoblasts can secrete the receptor activator of the nuclear factor-kappa-Β ligand (RANKL) which differentiates macrophages into osteoclasts. Targeting the osteoclast differentiation to modulate excessive bone resorption is an attractive strategy for the treatment of osteoporosis. There have been numerous studies to inhibit excessive bone resorption by osteoclasts [4].

Gold nanoparticles (GNPs) and silver nanoparticles (SNPs) have been utilized in a wide range of biomedical applications: drug delivery systems, bio-imaging devices, implants, optoelectronic devices, and prosthetics [5,6,7]. GNPs and SNPs could lead to new therapeutic strategies to improve bone regeneration or to treat bone disorders [6]. Specifically, GNPs and SNPs have been studied widely to promote differentiation into osteoblasts [8,9,10,11]. GNPs can inhibit osteoclast differentiation through the suppression of the MAPK and NF-κB signaling pathway [12]. Even though the inhibitory effects of differentiation into osteoclasts by the GNPs are extensively investigated [13,14,15], reports of the inhibitory effects of SNPs during the differentiation into osteoclasts are rare.

In this study, we isolated bone marrow-derived macrophages (BMMs) from Sprague–Dawley (SD) rats. The BMMs were then induced into osteoclasts for five days by adding RANKL and M-CSF. We compared the inhibitory effect of GNPs and SNPs on differentiation of BMMs into osteoclasts. We stained typical bone resorption factors, in this case, tartrate-resistant acid phosphatase (TRAP) and actin ring formation. Several genes related to osteoclasts were also measured using a quantitative real-time polymerase chain reaction (qRT-PCR). In addition, we investigated the signal pathways related to differentiation into osteoclasts by means of western blotting.

## 2. Materials and Methods

### 2.1. Materials

Chloroauric acid (HAuCl_4_) and trisodium citrate were purchased from Sigma Aldrich. (St. Louis, MO, USA), and 30 nm SNPs were purchased from Cytodiagnostics (Burlington; ON, Canada). The cell culture medium was purchased from GIBCO (Grand Island, NY, USA). Macrophage-colony stimulating factor (M-CSF) and RANKL were purchased from Peprotech (London, UK). The TRAP assay kit used here was purchased from Takara (Shiga, Japan). EZ-Cytox was purchased from Dogen (Seoul, Korea). All primers for Cathepsin K (CTSK), c-FOS, the nuclear factor of activated T-cells cytoplasmic 1 (NFATc1), TRAP, and glyceraldehyde 3-phosphate dehydrogenase (GAPDH) were purchased from Bioneer (Seoul, Korea). The antibodies used in this study were purchased from Invitrogen (Carlsbad, CA, USA).

### 2.2. Preparation and Characterization of the GNPs and SNPs

We created a HAuCl_4_ solution by mixing 136 mg of HAuCl_4_ powder with 800 mL of deionized water (DW). We also made a citrate solution by mixing 300 mg of citrate powder with 15 mL of DW. After the HAuCl_4_ solution was refluxed, the citrate solution was quickly added to it. After 15 min, the color of the solution changed to dark red. For an ultraviolet-visible (UV-Vis) spectroscopy analysis, the concentration of the GNP solution was adjusted until it was equal to the concentration of the SNP solution according to the Beer–Lambert law [16]. The Beer–Lambert law is used to calculate the molar concentrations of nanoparticles from measured absorbance and the molar extinction coefficient [17]. SNPs thirty nanometers (30 nm) in size were purchased from Cytodiagnostics (Burlington; ON, Canada). The sizes of the GNPs and SNPs were measured by dynamic light scattering (DLS, Malvern 4700, Malvern, Worcestershire, UK). The zeta potential (Zetasizer 2000, Malvern, Worcestershire, UK) levels were also measured to determine the surface charges of the GNPs and SNPs at a concentration of 0.1 nM.

### 2.3. Isolation of Bone Marrow-Derived Macrophages (BMM) and Primary Culture

The isolation protocol from the bone marrow of Sprague-Dawley (SD) rats developed by Kim et al. was used [18]. Here, the SD rats were euthanized by CO_2_ asphyxiation and the femur and tibia were collected aseptically. The bones were cut in half and placed in a 1.5 mL reaction tube. Bone marrow was collected by centrifugation (5 min, 5000× *g*). Cells were separated with a 40 μm cell strainer. Erythrocytes were lysed via a red blood cell lysing buffer (Sigma, St. Louis, MO, USA) for 30 s. The osteoclast differentiation protocol by Dia et al. was used [19]. The monocytes were seeded at 1.2 × 10^5^ cells on 48-well plates (Corning, NY, USA) and differentiated into macrophages, as follows. The cells were cultured with a culture medium (CM; alpha-minimum essential medium (α-MEM) containing 10% fetal bovine serum (FBS), 1% penicillin-streptomycin (P/S), and 20 ng/mL of M-CSF) for differentiation into BMMs for three days. The BMMs were then induced into osteoclasts for five days by adding RANKL to the CM. The CM with RANKL added to it is a complete culture medium (CCM) consisting of α-MEM containing 10% FBS, 1% P/S, 20 ng/mL of M-CSF, and 100 ng/mL of RANKL. The CCM was replaced with a fresh medium every two days. The multinuclear cells (MNCs) containing three or more nuclei were considered as osteoclasts. The isolation process was performed according to a protocol approved by the Institutional Animal Care and Use Committee (IACUC) of CHA University (IACUC200119).

### 2.4. Cytotoxicity Analyze

The cytotoxicity was evaluated at the various concentrations (0, 0.01, 0.05, 0.1, 0.2, 2, and 10 nM) of GNPs and SNPs. BMMs were seeded at 1.2 × 10^5^ cells on 48-well culture plates. After one day, the medium was changed, and a fresh medium was added containing a predetermined concentration of GNPs or SNPs. Cytotoxicity was measured using a cell counting kit (EZ-Cytox) at 48 h. The cells were washed with Dulbecco’s phosphate-buffered saline (DPBS). BMMs were replaced with fresh medium containing EZ-Cytox (500 μL of 0.1 mL/mL). After incubation for 2 h, the absorbance was measured at 450 nm with a microplate reader (Bio-Rad, Hercules, CA, USA). The absorbance of the 0 nM (without GNPs or SNPs) group at 48 h was fixed at 100% and the absorbance levels of the other groups were calculated relative to that level.

### 2.5. TRAP Assay and Measurements

BMMs were seeded at 1.2 × 10^5^ cells on 48-well culture plates and incubated with an osteoclast differentiation culture medium containing GNPs or SNPs. The negative control group (NC) was incubated without RANKL, and osteoclast differentiation was induced in the positive control group (PC) by M-CSF and RANKL. The osteoclasts were fixed by soaking in 4% paraformaldehyde for 15 min and washing three times with DPBS. The cells were placed in 0.1% Triton X-100 for 10 min at room temperature and washed three times with DPBS. Fixed cells were stained at 37 °C in the dark using the TRAP staining kit (Takara). After staining, TRAP-positive ^(+)^ cells were stained red. The stained cells were evaluated using a light microscope (Olympus IX71). The region of interests (ROIs, 1200 × 1000 μm, *n* = 4) were randomly designated. An area 1200 × 1000 μm in size was set as 100% and the stained area with TRAP^+^ was evaluated by the Image J program (National Institutes of Health (NIH), Bethesda, MD, USA).

### 2.6. Actin Ring Formation and Measurements

BMMs were seeded at 2 × 10^5^ cells on a confocal culture dish (SPL, Seoul, Korea). The control group was cultured with CM, and the RANKL group was cultured with CCM for 5 days. BMMs were differentiated into osteoclasts with CCM containing two concentrations (0.01 and 0.05 nM) of GNPs and SNPs. After five days, the cells were fixed by soaking in 4% paraformaldehyde for 15 min and washed three times with DPBS. The cells were placed in 0.1% Triton X-100 for 5 min at room temperature and washed three times with DPBS. Fixed cells were stained with rhodamine-conjugated phalloidin (1:400 dilution) at room temperature for 30 min and washed three times with DPBS. The actin ring formation process was observed using a fluorescence microscope (Olympus IX71). The ROIs (1200 × 1000 μm, *n* = 4) were also randomly designated.

### 2.7. Quantitative Real-Time Polymerase Chain Reaction (qRT-PCR)

To isolate the mRNA for the qRT-PCR process, BMMs (a density of 1.2 × 10^6^ cells) were seeded into six-well plates. The BMMs were differentiated into osteoclasts with CCM containing two concentrations (0.01 and 0.05 nM) of GNPs and SNPs. The cells were extracted using the Trizol reagent (Invitrogen) according to the manufacturer’s instructions at five days [13]. Complementary DNA (cDNA) was synthesized from 1 μg of total mRNA using a Maxim RT Premix kit (iNtRON Biotechnology, Inc., Korea). The qRT-PCR step was performed with an ABI Step-One real-time PCR system (Applied Biosystems, Warrington, UK). The reaction mixture consisted of the SYBR Green 2X PCR Master Mix (Applied Biosystems, Waltham, MA, USA), a cDNA template, and forward/reverse primers. The relative expression levels of TRAP, c-Fos, CTSK, and NFATc1 were normalized to that of GAPDH using the 2^−ΔΔCT^ method [20]. The primers of the measured mRNA genes were as follows: TRAP 5′-GCT GGA AAC CAT GAT CAC CT-3′ (sense) and 5′-GAG TTG CCA CAC AGC ATC AC-3′ (antisense); CTSK 5′- CTT CCA ATA CGT GCA GCA GA-3′ (sense) and 5′-TCT TCA GGG CTT TCT CGT TC -3′(antisense); c-Fos 5′-CAA GCG GAG ACA GAT CAA CTT G-3′ (sense) and 5′- TTT CCT TCT CTT TCA GCA GAT TGG-3′(antisense); NFATc1 5′-TGG AGA AGC AGA GCA CAG AC -3′ (sense) and 5′-GCG GAA AGG TGG TAT CTC AA -3′ (antisense); and GAPDH 5′- AAC TTT GGC ATT GTG GAA GG-3′ (sense), and 5′-ACA CAT TGG GGG TAG GAA CA -3′ (antisense) [21,22,23].

### 2.8. Western Blot

The BMMs (a density of 1.2 × 10^6^ cells) were seeded into six-well plates to isolate the protein for western blotting. The BMMs were differentiated into osteoclasts with CCM containing two concentrations (0.01 and 0.05 nM) of GNPs and SNPs. Cells were lysed with cold RIPA buffer containing protease (Roche Applied Science, Indianapolis, IN, USA) and phosphatase inhibitor cocktails (Sigma, St. Louis, MO, USA) [20,24]. The cells lysates were incubated on ice for 5 min and then centrifuged (10 min, 13,000× *g*). The supernatants were collected from each sample. Each concentration of the proteins was measured using a microplate reader (Bio-Rad) at a wavelength of 562 nm. The same amount of protein (30 μg) was congregated to 10% sodium dodecyl sulfate-polyacrylamide gel electrophoresis and transferred to nitrocellulose transfer membranes (Protran, Whatman, Germany). Each membrane was blocked with 5% bovine serum albumin for 1 h. The membranes were probed with primary antibodies against phosphorylated forms of the nuclear factor of kappa light polypeptide gene enhancer in a B-cell inhibitor, alpha (p-IκBα; 1:1000), p65(p-p65; 1:1000), extracellular signal–regulated kinase (p-ERK; 1:1000), c-Jun N-terminal kinase (p-JNK; 1:1000), p38 (p-p38; 1:1000), and NFATc1 (p-NFATc1; 1:1000). Subsequently, the membranes were stripped and reprobed with total forms of IκBα (t-IκBα; 1:1000), ERK (t-ERK; 1:1000AB_330744), JNK (t-JNK; 1:1000), and p38 (t-p38; 1:1000). As an internal control, β-actin was also probed into the membranes. An appropriate secondary antibody conjugated to horseradish peroxidase (1:5000 dilution) was then probed into the membranes. The visualized signal bands were detected using a horseradish peroxidase procedure with a ChemiDoc XRS system (Bio-Rad). The phosphorylated-form/total-form (p/t) volumes were calculated and quantified using the Image J program (NIH).

### 2.9. GNPs and SNPs Uptake Analysis Using Dark Field

The BMMs were seeded into 2 × 10^5^ cells on a confocal dish and cultured with α-MEM containing 10% FBS, 1% P/S, and 20 ng/mL of M-CSF for two days. The medium was changed, and a fresh medium was added containing 0.05 nM GNPs or 0.05 nM SNPs. Cells were incubated at 37 °C in humidified 5% CO_2_ for 24 h. The BMMs were washed with DPBS three times and immobilized with 4% paraformaldehyde at room temperature for 20 min. The fixed BMMs were rinsed with DPBS three times. The endocytosis particles were visualized using a 12-bit charge coupled device camera equipped with a special C-mount lens (Digital Imaging Systems, New Haven, CT, USA). The ROIs (1200 × 1000 μm, *n* = 4) were randomly designated within 100× magnification. The cell areas within the 100× images were set as 100%. The endocytosis outcomes of GNPs or SNPs into cells were measured by the Image J program (NIH).

### 2.10. Statistical Analyses

All values are presented as the mean ± standard deviation (SD). Tukey’s test was used to compare the two groups. Differences with *p* values for which “*” *p* < 0.05, “**” *p* < 0.01, and “***” *p* < 0.001 were considered statistically significant.

## 3. Results

### 3.1. Characterization of GNPs and SNPs

**Figure 1:**
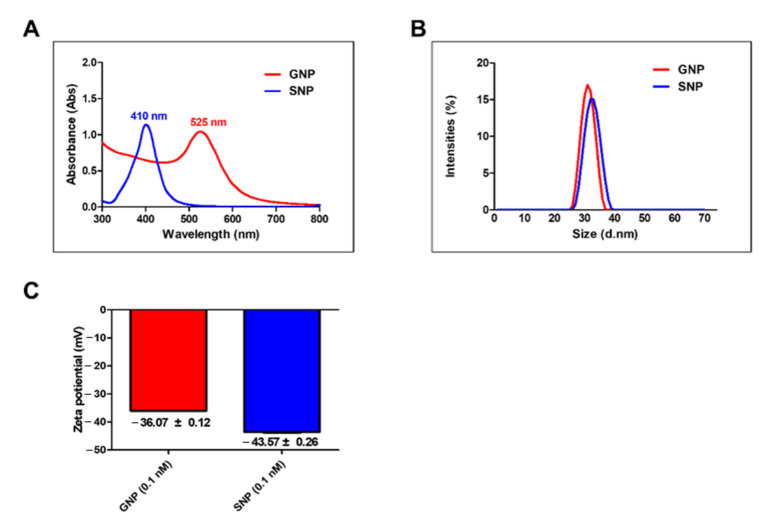
Characterization of gold nanoparticles (GNPs) and silver nanoparticles (SNPs). (**A**) Absorbance values ranged from 300 to 800 nanometer (nm) for the GNPs and SNPs. (**B**) Dynamic light scattering (DLS) analysis of the average diameter sizes of the GNPs and SNPs. (**C**) Zeta potential measurements of GNPs and SNPs.

To measure the sizes of the GNPs and SNPs, we used UV-Vis spectroscopy (Figure 1A). In the GNP group, the maximum absorbance was shown at 525 nm. In the SNP group, the maximum absorbance was at 410 nm. The diameter of the GNPs was 26–36 nm and the diameter of the SNPs was 27–38 nm (Figure 1B). GNPs and SNPs have different maximum absorbance wavelength each ranging from 400 to 800 nm and 200 to 800 nm, respectively [17,25]. The maximum absorbance wavelength of GNPs and SNPs increase in accordance with the size [17,26]. We confirmed that the sizes of the GNPs and SNPs were similar. The surface charges of the GNPs and SNPs were measured using the zeta potential (Malvern). The surface charge of the GNPs was −36.07 mV ± 0.12 and that of the SNPs was −43.57 mV ± 0.26 (Figure 1C). GNPs and SNPs have the surface charge around −40 mV.

### 3.2. Cell Viability by GNPs and SNPs in BMMs

**Figure 2:**
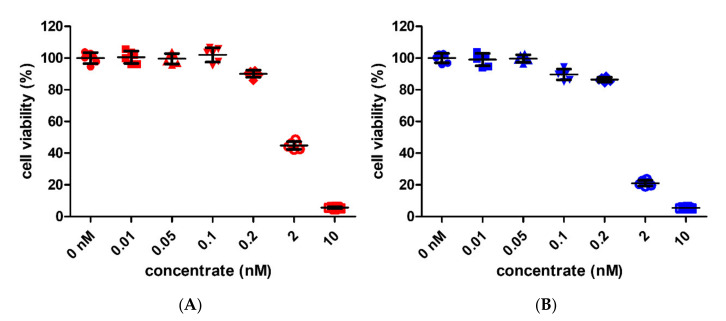
(**A**,**B**) Effect on the cell viability of bone-marrow-derived macrophages (BMMs). The BMMs were cultured with a culture medium (CM, without the RANKL medium) for two days. The concentrations of GNPs and SNPs were 0, 0.01, 0.05, 0.1, 0.2, 2, and 10 nM. The results are expressed as the mean ± standard deviation (SD, *n* = 6 per group).

The cell viability percentages for the GNPs exceeded 99% from 0 to 0.1 nM (0 nM: 100% ± 3.41, 0.01 nM: 100.56% ± 3.88, 0.05 nM: 99.6% ± 3.27, 0.1 nM: 102.02% ± 4.53, 0.2 nM: 90.08 ± 2.24, 2 nM: 44.84 ± 2.42, and 10 nM: 5.6 ± 0.58, Figure 2A). The cell viability percentages for the SNPs slightly decreased in the 0.1 nM concentration (0 nM: 100% ± 2.65, 0.01 nM: 99.06% ± 3.57, 0.05 nM: 99.67% ± 2.15, and 0.1 nM: 89.65% ± 3.15, 0.2 nM: 86.42 ± 1.53, 2 nM: 21.08 ± 1.76, and 10 nM: 5.4 ± 0.41, Figure 2B). In other words, the cell viability of SNPs was slightly inhibited at 0.1 nM. In addition, the cell viability of GNP and SNP groups dramatically decreased to 10% at 10 nM. Therefore, we compared the osteoclast inhibitory effects by GNPs and SNPs at sizes ranging from 0.01 and to 0.05 nM, which are not cytotoxic to BMMs.

### 3.3. GNPs and SNPs Inhibited the Formation of TRAP^+^ MNCs and Actin Rings

**Figure 3:**
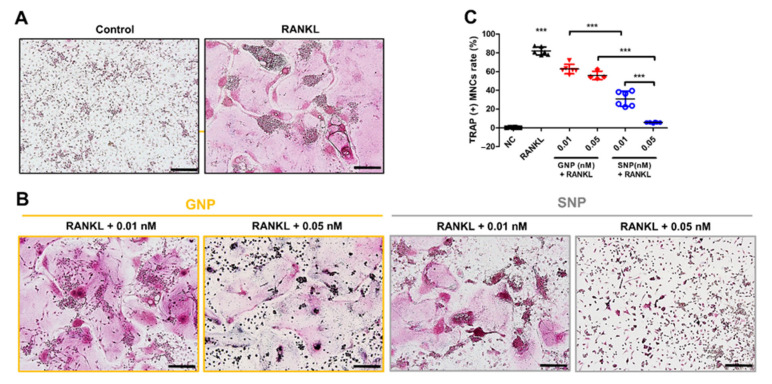
RANKL-induced tartrate-resistant acid phosphatase (TRAP) assay outcomes with GNPs or SNPs. BMMs were differentiated into osteoclasts with a complete culture medium (CCM, with RANKL medium) for five days. (**A**) BMMs of the control group were cultured with CM. BMMs of the RANKL group were cultured with CCM (without GNPs and SNPs). Scale bars: 200 μm. (**B**) TRAP+ multinuclear cells (MNCs) were stained at various concentrations of GNPs or SNPs. Scale bars: 200 μm. (**C**) The TRAP+ MNCs were quantified. The results are expressed as the mean ± SD (*n* = 6 per group). “***” indicates a significant difference for which *p* < 0.001.

**Figure 4:**
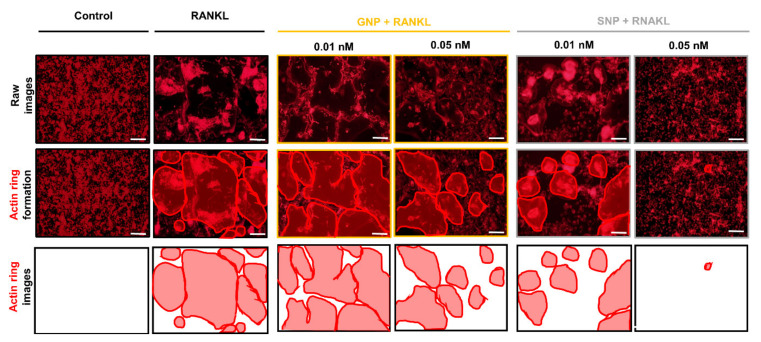
RANKL-induced actin ring structure with GNPs or SNPs for five days. BMMs were cultured at various concentrations of GNPs or SNPs. Scale bars: 200 μm. The results are expressed as the mean ± SD (*n* = 3 per group).

We evaluated the inhibitory effect by GNPs and SNPs during RANKL-induced osteoclast differentiation by means of TRAP staining (Figure 3). We confirmed noticeably differentiated osteoclasts from BMM by adding RANKL to CM (Figure 3A). TRAP^+^ MNCs were considered as osteoclasts. The levels of TRAP^+^ MNCs were 62.73% ± 5.04 and 55.9% ± 4.4 in the GNP groups (0.01 nM and 0.05 nM, respectively) and 30.92% ± 8.0 and 5.68% ± 0.5 in the SNP groups (0.01 nM and 0.05 nM, respectively, Figure 3B,C). These levels showed that the formation of TRAP^+^ MNCs in the SNP groups was significantly reduced compared with that in the GNP groups at an equal concentration (*** *p* < 0.001, Figure 3B,C). In addition, we confirmed the osteoclast morphology of the control group and RANKL group through actin ring staining (Figure 4). The actin ring formation in the SNP groups also was remarkably inhibited compared with that in the GNP groups at an equal concentration (Figure 4). Specifically, RANKL-induced actin ring formation was mostly inhibited at 0.05 nM in the SNP group (Figure 4). Taken together, the formation of TRAP^+^ MNCs with actin rings was inhibited most at 0.05 nM in the SNP group (Figure 3 and Figure 4).

### 3.4. Inhibited Gene Expression by GNPs and SNPs in the RANKL-Induced Osteoclasts

**Figure 5:**
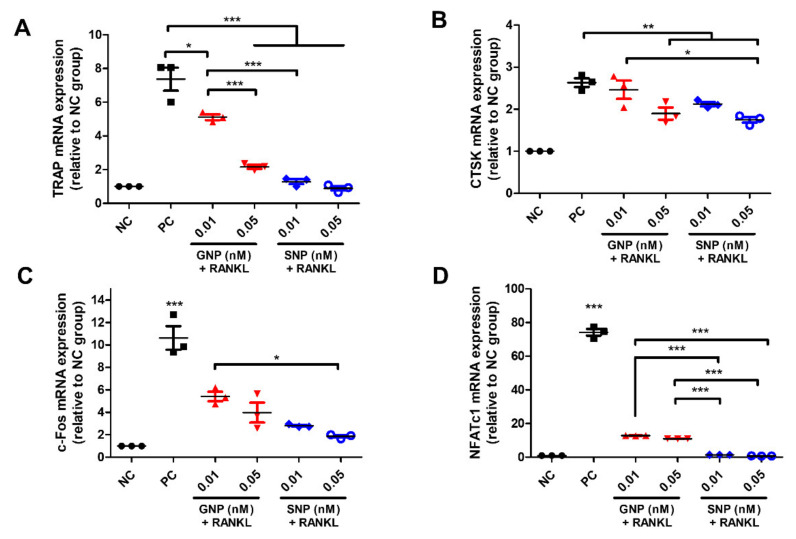
The mRNA expression level of (**A**) TRAP, (**B**) Cathepsin K (CTSK), (**C**) c-Fos, and (**D**) Nuclear activated T-cells, cytoplasmic 1 (NFATc1), with various concentrations of GNPs or SNPs. BMMs were differentiated into osteoclasts with CCM for five days. BMMs of the negative control (NC) group were cultured with CM. RANKL-induced BMMs of the positive control group (PC) were cultured with CCM. The mRNA expression levels at various concentrations of GNPs or SNPs were measured. The results are expressed as the mean ± SD (*n* = 3 per group). “*” indicates a significant difference for which *p* < 0.05. “**” indicates a significant difference for which *p* < 0.01. “***” indicates a significant difference for which *p* < 0.001.

We identified the inhibited gene expression by GNPs and SNPs in RANKL-induced osteoclasts using qRT-PCR. Among the groups, mRNA expression in the PC groups reached the maximum level (Figure 5). mRNA expression of TRAP, CTSK, c-Fos, and NFATc1 in the SNP groups was inhibited compared with that in the GNP groups at an equal concentration. mRNA expression was the inhibited most at 0.05 nM in the SNP group.

### 3.5. Effects of GNPs and SNPs in RANKL-Induced NF-κB Signal Pathways

**Figure 6:**
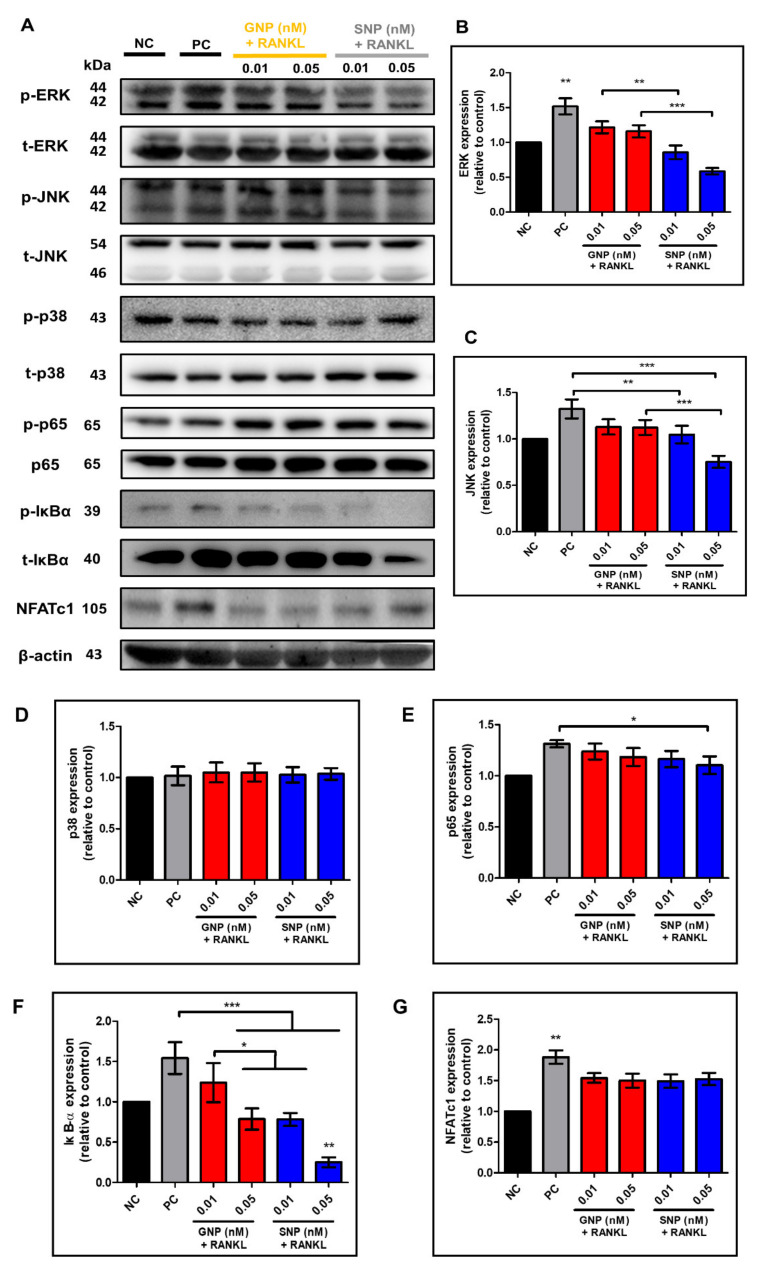
BMMs of the NC group were cultured with CM. BMMs were differentiated into osteoclasts with CCM for five days. The protein expression levels at various concentrations of GNPs or SNPs were measured. Immunoblotting was used to detect the phosphorylation (p) form and the total (t) form of ERK, JNK, p38, p65, and IκBα (nuclear factor of the kappa light polypeptide gene enhancer in the B-cell inhibitor, alpha). The phosphorylation (p) form of NFATc1 was examined by immunoblotting. (**A**) Representative band images of mitogen-activated protein kinase (MAPK) (ERK, JNK, and p38) and NF-κB (IκBα, p65) signal pathways, NFATc1, and β-actin. Quantitative analysis of (**B**) p-ERK/ERK, (**C**) p-JNK/JNK, (**D**) p-p38/p38, (**E**) p-p65/p65, (**F**) p-IκBα/IκBα, and (**G**) NFATc1/β-actin. Quantitative volumes are expressed as the mean ± SD (*n* = 3 per group). “*” indicates a significant difference for which *p* < 0.05. “**” indicates a significant difference for which *p* < 0.01. “***” indicates a significant difference for which *p* < 0.001.

We investigated the signal pathway in RANKL-induced osteoclasts by means of western blotting (Figure 6). The MAPK and NF-κB signaling pathways are two classical signaling pathways for the activation of osteoclasts [27]. We performed western blot assay to determine whether the GNPs and SNPs inhibit the MAPK and NF-κB signal pathways. As shown in Figure 6A, the p/t volume of ERK in PC group was 1.52 ± 0.12, whereas ERK in 0.05 nM SNP group was significantly lower than that in GNP group (*** *p* < 0.001, Figure 6B). The p/t volume of JNK in the 0.05 nM SNP group was also remarkably decreased compared with that in the 0.05 nM GNP group (*** *p* < 0.001, Figure 6C). Compared to the p/t volume of p38 in NC group, there were no significant differences observed in the other groups. The p/t volume of IκBα was 0.78 ± 0.07 at 0.01 nM in the SNP group (Figure 6F). The p/t volume of IκBα was 0.79 ± 0.11 at 0.05 nM in the GNP group. The p/t volume of IκBα showed the lowest levels at 0.05 nM in the SNP group (0.25 ± 0.05). The p/t volume of p65 was decreased in SNP group than that in PC group (NC: 1, PC: 1.31 ± 0.03, GNP 0.01 nM: 1.24 ± 0.06, GNP 0.05 nM: 1.18 ± 0.07, SNP 0.01 nM: 1.16 ± 0.07, and SNP 0.05 nM: 1.10 ± 0.07, Figure 6E). The expression of the transcription factor of osteoclast NFATc1 in GNP and SNP group was lower than that in PC group (NC: 1, PC: 1.88 ± 0.09, GNP 0.01 nM: 1.54 ± 0.06, GNP 0.05 nM: 1.5 ± 0.09, SNP 0.01 nM: 1.49 ± 0.09, and SNP 0.05 nM: 1.53 ± 0.08, Figure 6G).

The mean values of the internal control, β-actin, were 1.00 ± 0.00 (NC group), 1.01 ± 0.02 (PC group), 0.97 ± 0.01 (0.01 nM in the GNP group), 0.98 ± 0.01 (0.05 nM in the GNP group), 0.97 ± 0.01 (0.01 nM of SNP group), and 0.98 ± 0.02 (0.05 nM in the SNP group).

### 3.6. Comparison of the GNPs and SNPs Uptake Ratio in the BMMs

**Figure 7:**
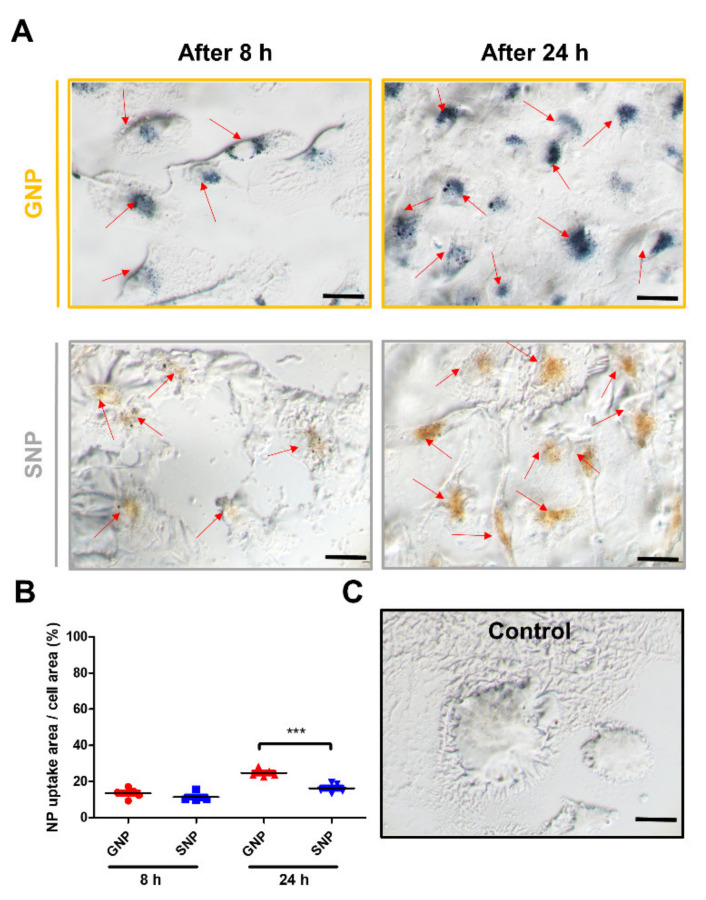
(**A**) Representative images of BMMs at 0.05 nM of GNPs or SNPs. Scale bars are 20 μm. (**B**) Quantitative volumes are expressed as the mean ± SD (*n* = 5 per group). “***” indicates a significant difference for which *p* < 0.001. (**C**) BMMs of the control group were cultured with CM. Scale bars are 20 μm.

We measured the amounts of uptake into the BMMs. BMMs were cultured with 0.05 nM of GNPs or 0.05 nM SNPs for 8 h and 24 h (Figure 7A,B). The uptake ratio of GNPs and SNPs in BMMs was increased at 24 h. The uptake ratios for the 8 h groups were 11.94 ± 4.26% and 10.2 ± 3.41% (GNPs and SNPs, respectively, Figure 7B). The uptake ratios for the 24 h groups were 26.67 ± 4.65% and 18.48 ± 5.2% (GNPs and SNPs, respectively, Figure 7B). The amount of uptake in the GNP group was significantly increased compared with that in the SNP group at 24 h (*** *p* < 0.001). GNPs and SNPs were not observed in the control group (Figure 7C).

## 4. Discussion

Osteoporosis patients have increased annually with rising life expectancy levels [4,28]. Specifically, the overexpression of osteoclasts induces low bone density in osteoporosis. Therefore, the inhibition of overexpression into osteoclasts is important for osteoporosis patients. GNPs have been promising candidates to inhibit differentiation into osteoclasts over the last few decades [13,29,30]. However, the inhibitory effects of SNPs are rarely reported. Even though Albers et al. reported the effect of osteoclast inhibition by SNPs, the only data presented consisted of optical densities using TRAP activity kits [31]. However, we evaluated the osteoclast inhibitory effect by SNPs through experiments involving TRAP and actin ring staining, qRT-PCR (Figure 3, Figure 4 and Figure 5). In addition, we demonstrated the inhibitory mechanism using western blot assays (Figure 6). We also compared the inhibitory effects by GNPs and SNPs at equal sizes and concentrations (Figure 3, Figure 4, Figure 5 and Figure 6).

The 30 nm GNPs are effective for the inhibition of osteoclast differentiation [15] and are useful with regard to their uptake into cells. Therefore, we used 30 nm particles in this study (Figure 1). GNPs and SNPs have different peak wavelengths according to their size [26]. The peaks at 525 and 410 nm demonstrated that the sizes of the GNPs and SNPs were 30 nm in terms of the mean diameter (Figure 1). SNPs have been used widely in anti-fungal and anti-inflammatory research [32,33,34] despite the fact that SNPs are quite toxic to cells [35,36]. Therefore, we investigated the inhibitory effects of osteoclasts at a concentration under the toxic range (Figure 2).

During osteoclast differentiation, cells became large, multinuclear cells. The differentiated osteoclasts stretch the actin filaments of the ring structures and tightly bind to the surrounding bone tissue [37,38]. These functions of osteoclasts were visually observed through TRAP and actin ring staining (Figure 3 and Figure 4). TRAP is abundantly expressed in osteoclasts and its expression indicates bone resorption [39]. CTSK is also secreted by osteoclasts, an enzyme that degrade collagen and other matrix proteins during bone resorption [40]. The functions of osteoclasts were also inhibited by GNPs and SNPs (Figure 3 and Figure 4).

The major transcription factors related to differentiation into osteoclasts are c-Fos and NFATc1 [37] The factors induce osteoclast differentiation from precursor cells. Specifically, the differentiated osteoclasts expel degrading enzymes such as CTSK and TRAP to break down the marrow cavity [1,4]. In this study, the levels of gene markers of TRAP, CTSK, c-Fos, and NFATc1 related to osteoclast differentiation were consistently lower in the SNP groups than in the GNP groups (Figure 5).

RANKL-induced BMMs activate MAPK and NF-κB (nuclear factor kappa-light-chain-enhancer of activated B cells) signals and cause osteoclast differentiation. MAPK signal is induced by the phosphorylation of ERK, JNK, and P38 in RANKL-induced osteoclasts [41]. In the NF-κB pathway, IκBα inhibits the initiation of the NF-κB signal pathway. However, IκBα is phosphorylated by RANKL. In other words, the phosphorylation of IκBα leads to osteoclast differentiation [40,41,42]. GNPs slightly inhibited RANKL-induced phosphorylation of ERK, JNK, IκBα, and p65, whereas SNP groups further inhibited the phosphorylation (Figure 6).

We compared the amounts of cellular uptake in the GNP and SNP groups. As shown in Figure 7, the amounts in the GNP groups were higher than those of the SNP groups for 8 h and 24 h. The charge of the cell membrane is negative [42]. In negatively charged nanoparticles, the lower the surface charges, the more increased the amounts of cellular uptake [43,44]. In this study, the surface charges of SNPs were lower than those of the GNPs (Figure 1C). This lower charge in SNPs may inhibit cellular uptake [43,45,46,47,48]. Nevertheless, the levels related to osteoclast differentiation were consistently lower in the SNP groups than in the GNP groups, as mentioned above.

Bai et al. demonstrated that GNPs in osteoclasts inhibited the acidification of surrounding environment [49]. Acidic environment promotes osteoclast differentiation. Consistent with previous reports, TRAP expression was decreased in GNP-treated osteoclasts (Figure 3).

## 5. Conclusions

Our study suggests that SNPs can be a useful means of inhibiting osteoclast differentiation and that they are applicable as a treatment modality for osteoporosis patients.

## Data Availability

The data used to support the findings of this study are included within the article.
